# Efficacy of antibacterial-loaded coating in an in vivo model of acutely highly contaminated implant

**DOI:** 10.1007/s00264-013-2237-2

**Published:** 2013-12-22

**Authors:** Gianluca Giavaresi, Enzo Meani, Maria Sartori, Andrea Ferrari, Davide Bellini, Anna C. Sacchetta, Joachim Meraner, Andrea Sambri, Caterina Vocale, Vittorio Sambri, Milena Fini, Carlo L. Romanò

**Affiliations:** 1 Laboratory of Preclinical and Surgical Studies, Rizzoli Orthopedic Institute, Bologna, Italy; 2 Laboratory of Biocompatibility, Technological Innovations and Advanced Therapies, Department Rizzoli, RIT- Rizzoli Orthopedic Institute, Bologna, Italy; 3 NOVAGENIT SRL, Mezzolombardo, Trento Italy; 4 DMCSS - Microbiology, University of Bologna, St. Orsola Hospital, Bologna, Italy; 5 Unit of Microbiology, Central Laboratory, Area Vasta Romagna-AUSL, Pievesestina, Cesena, Italy; 6 San Siro Clinic Institute– San Donato Group, Milan, Italy; 7 Centre for Reconstructive Surgery and Osteo-articolar Infections, Galeazzi Orthopedic Institute, Milan, Italy; 8 Laboratory of Preclinical and Surgical Studies, Istituto Ortopedico Rizzoli, Via Di Barbiano, 1/10, 40136 Bologna, Italy

**Keywords:** Infection, Implant, Coating, Nail, Prosthesis, Hydrogel, DAC

## Abstract

**Purpose:**

The purpose of this study was to test the ability of DAC®, a fast resorbable, antibacterial-loaded hydrogel coating, to prevent acute bacterial colonization in an in vivo model of an intra-operatively highly contaminated implant.

**Methods:**

A histocompatibility study was performed in 10 adult New Zealand rabbits. Then, methicillin-resistant *Staph. aureus* were inoculated in the femur of 30 adult New Zealand rabbits at the time of intra-medullary nailing; vancomycin-loaded DAC® coated nails were compared to controls regarding local and systemic infection development.

**Results:**

Histocompatibility study showed no detrimental effect of DAC® hydrogel on bone tissue after 12 weeks from implant. After seven days from implant, none of the rabbits receiving vancomycin-loaded DAC® nail showed positive blood cultures, compared to all the controls; vancomycin-loaded DAC® coating was associated with local bacterial load reduction ranging from 72 to 99 %, compared to controls.

**Conclusions:**

Vancomycin-loaded DAC® coating is able to significantly reduce bacterial colonization in an animal model of an intra-operatively highly contaminated implant, without local or general side effect.

## Introduction

Post-surgical infections represent a common healthcare problem, with *Staphylococci* being the leading micro-organism involved [1, 2].

According to the latest Annual Epidemiological Report drawn in 2012 by the European Centre for Disease and Control, a successful control has been performed, and especially *Methicillin-resistant Staphylococcus aureus* (MRSA) isolation appears stable or decreasing in many European Countries [3]. Nevertheless, in some of them, the percentage of MRSA isolation is above 25 %, suggesting how neither the wide range of antimicrobial agents nor the best clinical prophylaxis in the operating room led to a complete surgical infection control and eradication. Concerning the orthopedic field, the issue posed by bacterial infection is considerable, especially because MRSA is among the most frequently reported agent associated with orthopaedic implant infection [4].

In situations in which an inert foreign body is implanted into an already injured and weakened tissue, a competition develops for the colonization of the implant surface between bacteria and the cells of the immune system. However, bacteria have the advantage over immune system cells of faster reproductive processes and an extreme flexibility in adapting to the environment [5]. While infection after primary joint replacement in normal hosts is relatively rare, occurring in less than 2 % of the patients, septic complications may be much more frequent in immunocompromised hosts or after revision or contaminated surgery [6]. In spite of the relatively low incidence of post-surgical septic complications, their overall socio-economic burden is going to increase, due to various factors [7].

Different treatment strategies can be adopted to counteract orthopaedic infections, depending on overall patient conditions (immune system, joint status and its functionality, etc.) and according to involved pathogens and infection severity [8]. According to widely available data, local antibiotic delivery represents an effective strategy to prevent and even to treat bone infections [9–12]. Polymethyl-methacrylate (PMMA) bone cement has probably been the most widely adopted material to locally release antibiotic, although it may have some drawbacks, including its non-biodegradability, possible microbial adhesion and biofilm formation, restricted range of loadable antibiotics and long lasting release with possible increase of antibiotic resistance [13, 14]. Due to these drawbacks and to the widespread use of cementless implants in orthopedic and trauma surgery, the attention is gradually shifting towards alternative delivery devices, and considerable attention is now being addressed to active biodegradable materials that offer the advantage of bioreabsorption, reducing reactions induced by foreign bodies and increasing total local release of the drugs [15].

Hydrogels represent promising and potential alternative materials able to locally convey antibiotics and regulate bone bacterial infections related to orthopaedic devices [16, 17].

The aim of the present study was to test the ability of an antibacterial-loaded bioreabsorbable hydrogel coating, obtained by derivatization of low molecular weight hyaluronic acid (HA) with poly-D,L-lactic acid (PDLLA) [18], to reduce bacterial acute colonization in an animal model of intra-operative high-load bacterial contamination of an implant.

## Materials and methods

### Hydrogel synthesis and characterization

The study was performed on a fully bio-absorbable patented hydrogel (DAC®, Novagenit®, Mezzolombardo, Italy; Patent no.: WO2010/086421 A1), recently CE marked, which is provided as a sterile powder in a prefilled syringe to be hydrated at the time of use. The reconstitution takes place in minutes and only a few steps are sufficient to obtain a highly adhesive hydrogel with a rheological viscosity of 150–400 Pa·s ready to be easily spread on the surface of an orthopedic implant by means of a specifically designed spreader. According to product specifications, reconstitution of the hydrogel can be carried out by mixing it with various antibacterial compounds.

### Vancomycin release studies

Release studies were performed with DAC® hydrogel loaded with 2 % (w/v) of vancomycin. Briefly, approximately 210 ± 20 mg of hydrogel were homogeneously spread onto sandblasted titanium disk (Adler Ortho Srl, Cormano-Milan, Italy) having a diameter of 25 mm and the exact quantity was determined by means of an analytical balance. The disks with the applied hydrogel were then vertically placed into 50 ml centrifuge tubes (Corning®, MA, USA) and covered with 6 ml of Dulbecco’s phosphate buffered saline (Sigma®, Milan, Italy). Tightly closed centrifuge tubes were incubated at 37 %, 85 % RH without agitation. At defined intervals (i.e. at two, four, eight and 24 hours) 1 ml samples were aseptically taken, placed into 2 ml cryogenic tubes (Corning®, MA, USA) and frozen at –20 °C until analysis. Sample volume was immediately replaced by fresh, pre-warmed phosphate buffer in order to keep the volume constant throughout the entire study.

Release data were calculated as follows: analytical raw data (expressed as μg/ml) normalized to the instrumental standard curve were multiplied with the total buffer volume (ml) used for incubation in order to determine substance quantity (μg). Since sample volume was replaced with fresh buffer in order to keep the volume constant, and this unavoidably leads to a gradual dilution, the overall quantity (in μg) for a given time point was determined by adding to the analytically determined amount the substance quantities removed in the previous sampling points. Finally, the substance release was expressed as the percentage of the total quantity initially loaded (i.e. concentration of the substance inside the hydrogel [μg substance/μg hydrogel] × quantity of hydrogel loaded on the disc [μg]).

### In vivo studies

The animal research protocols were approved by the Ethical Committee of the Rizzoli Orthopedic Institute and by the appropriate public authorities and carried out in accordance with the European and Italian Law on animal experimentation. The experiments included an in vivo evaluation of hydrogel long-term histocompatibility, according to ISO 10993-6: 2009 standards and a test of its anti-infective capabilities in a model of acute, high-load intra-operative bacterial contamination of an intra-medullary implant.

Forty adult male New Zealand pathogen free rabbits (Harlan Laboratories SRL, S.Pietro al Natisone, Udine), 2.7 ± 0.2 kg b.w., were housed in individual cages and fed with a standard pellet diet (Mucedola, Settimo Milanese, Milan, Italy) and water ad libitum. The animals were divided into seven groups as reported in Table 1. All surgical procedures were performed under general anaesthesia induced by intramuscular injection of 44 mg/kg ketamine (Imalgene 1000, Merial Italy S.p.A, Assago-Milan, Italy) and 3 mg/kg xylazine (Rompun, Bayer SpA, Milan, Italy). Anaesthesia was maintained with O_2_/air (1/0.4 l/min) mixture and 2–2.5 % sevorane (Sevorane, ABBOTT Srl, Latina, Italy).

**Table 1:** In vivo experimental set-up

Group	*N*	Treatment		Experimental time
Histocompatibility evaluation				
A	10	Right Femur	Left femur	12 weeks
		DAC hydrogel	Sodium hyaluronate	
Perimplant MRSA bone infection study				
MRSA inoculum in right femur			Treatment	
1	5	High load (10^6^ CFU)	0 % Vancomicyn-loaded DAC®	7 days
2	5		2 % Vancomicyn-loaded DAC®	
3	5		5% Vancomicyn-loaded DAC®	
4	5	Low load (10^4^ CFU)	0 % Vancomicyn-loaded DAC®	
5	5		2 % Vancomicyn-loaded DAC®	
6	5		5% Vancomicyn-loaded DAC®	

Postoperatively, analgesic therapy was administered for three days (0.1 ml/kg/day metamizole sodium; Farmolisina, Ceva Vetem SpA, Monza-Brianza, Italy) and antibiotics were administered differently according to experimental designs (Table 1):

- Histocompatibility study: Group A (*n* = 10) underwent a systemic antibiotic therapy with i.m. injections of 0.6 ml/kg flumequil (Flumexil, FATRO SpA, Bologna, Italy) for three days.
- Perimplant MRSA bone infection study: Groups 1–6 (*n* = 5 for each group) underwent a systemic antibiotic therapy with i.v. injections of 2 % vancomycin in two subsequent administrations, namely, 0.8 ml at one hour before MRSA inoculum and intramedullary nail implant and 0.8 ml within 24 hour from the inoculums.

At the end of experimental times, animals were pharmacologically euthanised with the intravenous administration of 1 ml of Tanax (Tanax, Hoechst Roussel Vet, Milan, Italy) under general anaesthesia as previously reported.

#### Histocompatibility study

Under sterile surgical conditions in group A, parapattelar skin incision was performed bilaterally onto femoral condyles, anterior cruciate ligament was displaced and articular surface was exposed. A small stab incision was then performed and 2.5-mm drill holes were created in the intercondylar region of each femur. Through this aperture, a 16-gauge needle was introduced to the medullary cavity and 0.5 ml of bone marrow was aspirated. Subsequently 1 ml of DAC® hydrogel was slowly injected in the right condyles while 1 ml of sodium hyaluronate (HYALGAN®, 20 mg/2 ml Fidia Farmaceutici SpA; Abano Terme, Padova, Italy) as control material was injected in the left condyles. Finally, the defects created in the intercondylar region were sealed with sterile bone wax to prevent leakage of the injection (Knochenwachs, B Braun, Aesculap AG & Co., KG, Tuttlingen, Germany) and wounds were sutured in layers.

Twelve weeks after surgery, femurs were explanted and during dissection the presence of clinical signs of inflammation, haematomas, oedema, or tissue reactions were macroscopically evaluated; also lymphatic draining district (popliteal and inguinal lymph nodes) was taken into consideration. Cylindrical bone segments about 1-cm long were obtained with an oscillating saw by femoral diaphyses. Distal femoral epiphyses and cylindrical diaphysis segments were fixed in 4 % paraformaldehyde.

#### Acute intra-operative MRSA implant contamination study

The MRSA strain used in this study was originally isolated from a patient suffering from chronic osteomyelitis and maintained in culture for several years. The procedure adopted to isolate and characterize the antibiotic resistance of the MRSA strain used has already been reported [10]. Briefly, the MRSA inoculum was prepared from overnight cultures grown in Mueller Hinton Broth (MHB) starting from a frozen batch at –80 °C and aliquots were prepared. The cells were harvested by centrifugation, washed with saline solution and resuspended in order to obtain a final density of 5x10^6^ or 5×10^4^ colony-forming units (CFU)/ml of MHB, respectively. The MRSA inocula were stored at 4 °C and used within 12 hours of preparation. The density and purity of each bacterial preparation were verified before the surgical session by performing colony counts on selective media after plating on horse blood agar. The number of colonies in each plate was counted by a blinded operator and the bacterial concentration was determined.

Under sterile surgical conditions, a small stab incision and a progressively larger drill hole up to 3.5 mm in diameter was created in the intercondylar region of the right femur of each rabbit. Subsequently, a 18-gauge needle was inserted until the medullary cavity and 0.2 ml of bacterial suspension, containing 10^6^ or 10^4^ CFU of MRSA respectively, was directly inoculated according to the experimental group reported in Table 1. Immediately after inoculation, sandblasted titanium nails (Adler Ortho Srl, Cormano-Milan, Italy), 3 mm in diameter and 40 mm in length with a surface roughness (Ra) of approximately 7 μm (standard reference, Ra = 5–9 μm) were coated directly by the surgeon before implantation with DAC®hydrogel loaded with 0 %, 2 % or 5 % (w/v) vancomycin (Vancomycina Hospira 500 mg, Hospira SpA, Milan, Italy) respectively and implanted through the same surgical access. Finally, the created defect in the intercondylar region was filled with a small block of bone wax (Knochenwachs, B Braun, Aesculap AG & Co., KG, Tuttlingen, Germany) to avoid bacterial inoculums and hydrogel spilling out.

Seven days after surgery, under general anaesthesia, the animals were submitted to 20 ml intracardiac blood sampling for aerobic and anaerobic cultures (BacT/ALERT® 3D, BioMérieux, Marcy l’Etoile, France). After euthanasia and under sterile surgical conditions, the right femurs of each rabbit were explanted, and the intramedullary nails were removed in order to perform a swab of the femurs for microbiological analysis. Thereafter, a diaphyseal cortical segment of approximately 1 cm was obtained for further microbiological investigations.

### Histological and histomorphometric investigation

Bone samples retrieved by group A were immediately dehydrated and finally embedded in a methyl methacrylate resin solution (Merck Schuchardt OHG, Hohenbrunn, Germany). Transverse sections of femoral diaphysis were obtained by using rotative microtome (Leica SP1600, Leica Microsystems SpA, Milan, Italy), while an automatic cutting grinding system (EXAKT, EXAKT Apparatus GmbH, Norderstedt, Germany) was used to obtain longitudinal sections of femoral epiphysis. The obtained sections were then ground and polished to 20 ± 10 μm thickness and finally stained with toluidine blue, acid fuchsin and fast green.

All histological sections were digitized adopting Aperio ScanScope CS System (Aperio Technologies, Vista, CA, USA) at full resolution (1781 × 1467 pixels). Longitudinal epiphyseal sections were used to perform a qualitative histological evaluation of cellular or tissue alterations related to DAC® hydrogel or HYALGAN® control material injection. Instead, sections related to femoral mid-diaphysis were used to perform histomorphometric investigations by means of the image analysis system Leica Qwin (Leica Microsystems Ltd). The following histomorphometric parameters were evaluated onto sections:

- Diaphyseal sectional area (Se.Ar, mm²): area of the section comprising the diaphyseal portion of cortical bone tissue and the medullary area
- Medullary area (Me.Ar, mm²): cross-sectional area of the diaphyseal medullary cavity
- Cortical area (Ct.Ar, mm²): total area of cortical bone obtained by applying the following formula: Se.Ar-Me.Ar
- Cortical bone thickness (mm): distance between the endosteal and periosteal surface of the cortical bone measured in ten different points distributed onto the entire diaphyseal section

### Microbiological investigations

Microbiological investigations were carried out on specimens collected by groups 1 to 6. The blood culture procedure was performed following a previously reported standard protocol [19]. The microbiological investigations were performed within two hours. Briefly, 1 ml of MHB was added to each swab while the diaphyseal cortical segments and the intramedullary nails were individually placed into 3 ml of antibiotic free MHB. All samples were then agitated for three minutes on a vortex to resuspend collected bacteria. At the end of this procedure, a serial ten-fold dilution of each suspension, i.e. from swabs and cortical bone segments, was prepared to determine the bacterial load. Briefly, 10 μl of each dilution was plated onto horse blood agar and incubated at 37 °C for 48 hours. At the end of the incubation period the number of MRSA colonies on each plate was counted and the total viable CFU load was determined and expressed as log CFU/ml.

### Statistical analysis

Statistical evaluation of data was performed using the software package SPSS 12.1 (SPSS Inc., Chicago, IL USA). Data are reported as median (min-max) at a significance level of *p* < 0.05. After having verified the normal distribution and the homogeneity of the variance, the non-parametric Kruskal-Wallis test, followed by the Monte Carlo methods to compute probability, was used to analyse data. Then, Mann-Whitney U was performed to detect significant differences between groups by adopting Bonferroni correction.

## Results

### Release studies

The functionality of DAC® hydrogel to efficaciously and efficiently release previously loaded vancomycin was determined in vitro.

**Fig. 1:**
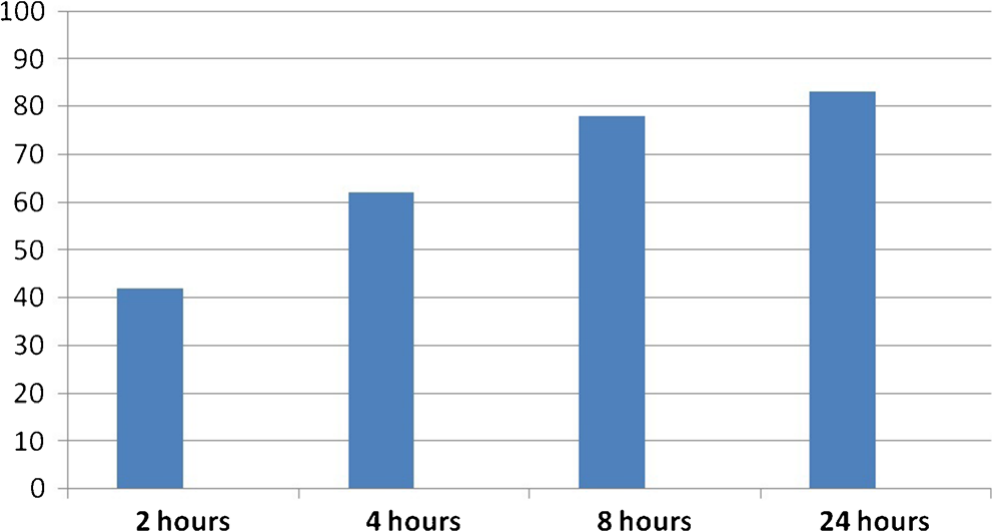
Release of vancomycin from DAC® hydrogel at defined intervals during incubation at 37 °C. Release expressed as percentage of total antibiotic quantity loaded

As shown in Fig. 1, release studies carried out on three distinct production batches of DAC® product demonstrated that already after two hours from incubation start approximately 40 % of the overall vancomycin quantity were released into the surrounding solution reaching more than 80 % within the first 24 hours.

### Histocompatibility study

**Fig. 2:**
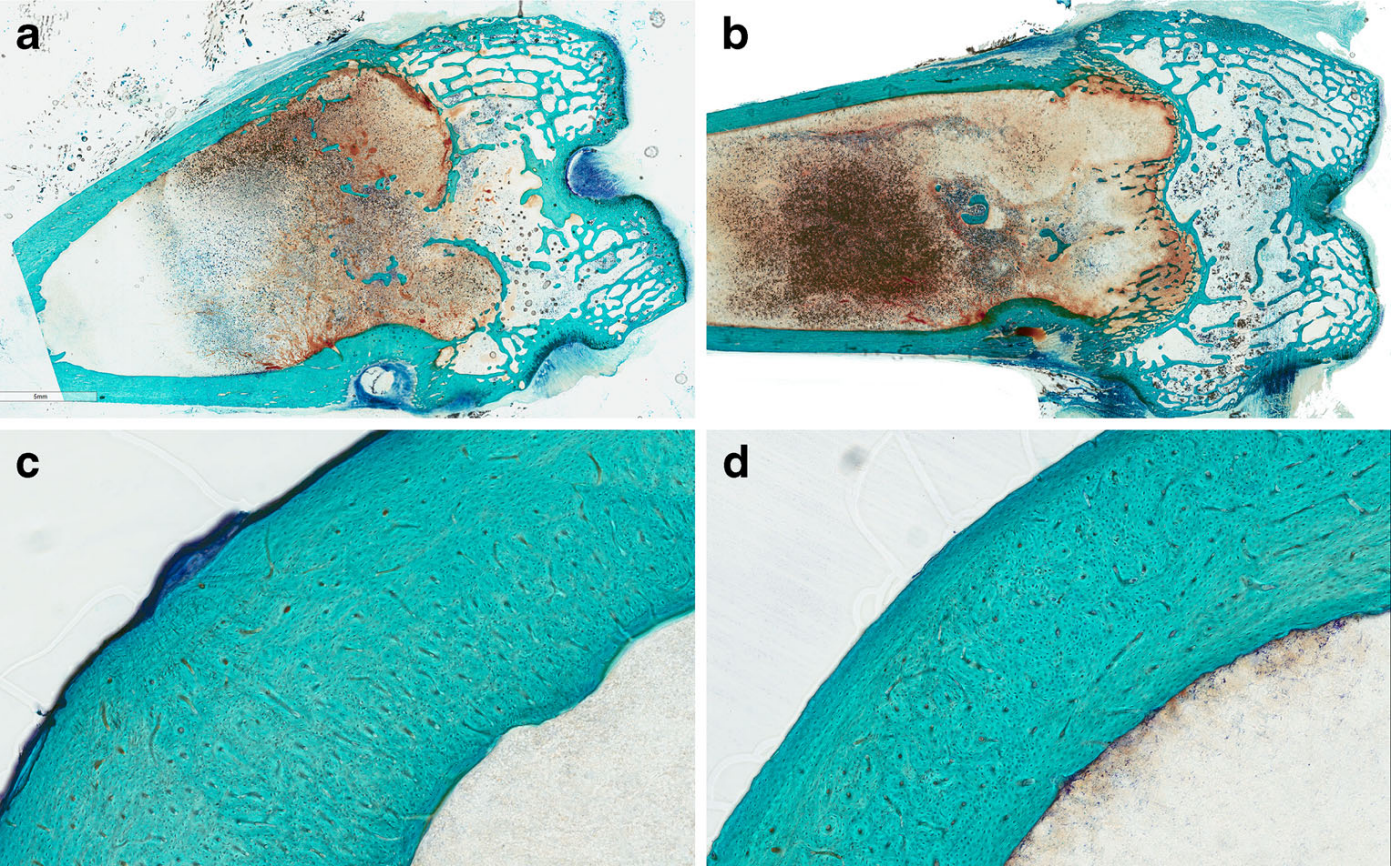
Histological images of femoral distal epiphyses (**a**, **b**) and cortical diaphyses (**c**, **d**) treated with DAC® (**a**, **c**) and control (**b**, **d**) hydrogel (HYALGAN®) at a magnification of 0.5x (**a**, **b**) and 5x (**c**, **d**). Toluidine blue, acid fuchsin and fast green

Neither inflammatory nor degenerative signs were found in femoral segments and joint surfaces at the retrieval. The observation of popliteal and inguinal lymph nodes showed no signs of swelling or morphological alteration due to inflammatory or degenerative processes. No differences in the structural organization of bone tissue between segments treated with DAC® and control hydrogel were observed (Fig. 2). The histomorphometric evaluations showed no statistically significant differences between DAC® and control hydrogel for all parameters evaluated (Table 2), confirming the qualitative evaluation.

**Table 2:** Histomorphometric results of cortical bone parameters of group A. Mean ± SD, *n* = 10

Cortical bone parameters	Right femoral middiaphysis DAC hydrogel	Left femoral middiaphysis Sodium hyaluronate
Se.Ar (mm²)	166.99 ± 25.74	170.26 ± 28.75
Me.Ar (mm²)	88.78 ± 17.38	91.97 ± 25.90
Ct.Ar (mm²)	78.21 ± 15.75	78.29 ± 13.47
Ct.Th (mm)	2.15 ± 0.54	2.01 ± 0.41

*Se.Ar* sectional area, *Me.Ar* medullary area, *Ct.Ar* cortical area, *Ct.Th* cortical thickness

### Perimplant MRSA bone infection study

All the animals tolerated surgery and the post-operative period well. Microbiological investigations showed high bacterial load in blood cultures taken in control animals, while blood cultures of vancomycin-loaded hydrogel groups were all negative (Table 3) (*p* < 0.05).

**Table 3:** Microbiological results of blood expressed as CFU/ml of 0 %, 2 % and 5 % (w/v) vancomycin-loaded DAC® treated groups for both MRSA bacterial loads (10^4^ or 10^6^ CFU): median (min-max), *n* = 5

Group	MRSA load	Treatment	Median (Min – Max)
1	High load (10^6^ CFU)	0 % Vancomicyn-loaded DAC®	1.00E + 07 (0.00E +00 – 1.00E + 07)
2		2 % Vancomicyn-loaded DAC®	0.00E + 00
3		5% Vancomicyn-loaded DAC®	0.00E + 00
4	Low load (10^4^ CFU)	0 % Vancomicyn-loaded DAC®	5.00E + 03 (0.00E +00 – 5.00E + 03)
5		2 % Vancomicyn-loaded DAC®	0.00E + 00
6		5% Vancomicyn-loaded DAC®	0.00E + 00

Mann-Whitney U test: group 2 (*p* = 0.032) and group 3 (*p* = 0.032) versus group 1, and group 5 (*p* = 0.009) and group 6 (*p* = 0.024) versus group 4

Intra-medullary swabs (Table 4) and bone microbiological samples (Table 5) were positive for MRSA at one week from surgery ranging from 10^3^ to 10^8^ CFU/ml. In groups 1 and 4, in which antibiotic-free DAC® hydrogel was applied together with a high (10^6^) or lower (10^4^) MRSA inoculum, respectively, bacterial contamination increased up to more than 10^8^ in group 1 and 10^5^ CFU in group 4 after one week from surgery, even in the presence of vancomycin administered systemically. On the contrary in groups 2 and 3, application of DAC® hydrogel loaded with 2 % or 5 % (w/v) of vancomycin, respectively, in the presence of a high MRSA inoculum (i.e. 10^6^ CFU) resulted in a significant (*p* < 0.05) inhibition of MRSA growth at both antibiotic concentrations (2 % and 5 %), reducing the local load in all the sites of investigation by about 99 % in comparison to group 1. Similar results were obtained for groups 5 and 6 in comparison to group 4 where, in the presence of a lower (10^4^ CFU) MRSA load, significant (*p* < 0.05) reductions were achieved by the application of vancomycin loaded DAC® hydrogel: 72–90 % for intramedullary swabs in groups 5 and 6, respectively.

**Table 4:** Microbiological results of intramedullary swab expressed as CFU/ml of 0 %, 2 % and 5 % (w/v) vancomycin-loaded DAC® treated groups for both MRSA bacterial loads (10^4^ or 10^6^ CFU): median (min-max), *n* = 5

Group	MRSA load	Treatment	Median (Min – Max)
1	High load (10^6^ CFU)	0 % Vancomicyn-loaded DAC®	1.00E + 08 (7.40E + 05 – 1.07E + 08)
2		2 % Vancomicyn-loaded DAC®	5.00E + 04 (7.40E + 03 – 8.40E + 04)
3		5% Vancomicyn-loaded DAC®	4.80E + 04 (8.80E + 03 – 8.40E + 04)
4	Low load (10^4^ CFU)	0 % Vancomicyn-loaded DAC®	5.00E + 05 (3.20E + 04 – 5.00E + 05)
5		2 % Vancomicyn-loaded DAC®	1.40E + 04 (5.00E + 03 – 3.90E + 04)
6		5% Vancomicyn-loaded DAC®	4.90E + 03 (2.80E + 03 – 5.00E + 04)

Mann-Whitney U test: group 2 (*p* = 0.012) and group 3 (*p* = 0.009) versus group 1; Group 5 (*p* = 0.027) and group 6 (*p* = 0.048) versus group 4

**Table 5:** Microbiological results of cortical bone expressed as CFU/ml of 0 %, 2 % and 5 % (w/v) vancomycin-loaded DAC® treated groups for both MRSA bacterial loads (10^4^ or 10^6^ CFU): median (min-max), *n* = 5

Group	MRSA load	Treatment	Median (Min – Max)
1	High load (10^6^ CFU)	0 % Vancomicyn-loaded DAC®	1.00E + 08 (6.50E + 05 – 1.00E + 08)
2		2 % Vancomicyn-loaded DAC®	5.90E + 04 (7.20E + 03 – 8.70E + 04)
3		5% Vancomicyn-loaded DAC®	4.80E + 04 (7.90E + 03 – 9.50E + 04)
4	Low load (10^4^ CFU)	0 % Vancomicyn-loaded DAC®	5.00E + 05 (4.50E + 05 – 7.80E + 05)
5		2 % Vancomicyn-loaded DAC®	4.60E + 05 (3.50E + 05 – 6.70E + 05)
6		5% Vancomicyn-loaded DAC®	1.90E + 04 (8.00E + 02 – 4.40E + 04)

Mann-Whitney U test: group 2 (*p* = 0.009) and group 3 (*p* = 0.012) versus group 1; group 6 versus group 4 (*p* = 0.009) and group 5 (*p* = 0.012)

## Discussion

Biofilm-related infections are among the main reasons for failure of joint prosthesis with high associated social and economical costs [20, 21], often requiring challenging diagnosis and prolonged and complex treatments [22–24].

Although antibacterial coatings have been proposed to prevent bacterial colonization and biofilm formation, available technologies are far from large scale application, due to various limitations, including questionable long-term effect on bacterial resistance, osteointegration, regulatory issues and costs [25, 26].

A first aim of the present study was to provide further analysis concerning histocompatibility of DAC® hydrogel that already met ISO standards, according to previous studies (Novagenit SRL, data on file); in this regard, the present research shows no long-term effect of DAC® hydrogel on bone tissue.

A second aim was to evaluate the antibacterial effect of the hydrogel, in combination with vancomycin in an animal model. In line with previous observations that bacterial adhesion and subsequent biofilm formation develop within hours after biomaterials implant into the human body, when a “race to the surface” takes place between the host’s cells and the colonizing bacteria eventually present at the surgical site [12, 27–29], this study provides evidence that local application of a resorbable hydrogel coating and a fast local delivery of antibiotics is effective in significantly reducing bacterial colonization of intra-medullary cavity and bone in an animal model. Current data show that, in the presence of high load bacterial contaminations (10^6^ and 10^4^), as used in this study, even targeted systemic prophylaxis is not able to prevent local infection or systemic spreading of bacteria in a model of implant-related infection in a not-immunocompromised host. Local antibacterial coating did, on the other hand, completely abolish haematogenous spreading of bacteria after local inoculums and dramatically reduced the bacterial load at the bone/implant interface.

This study has some limits:

- The antibacterial effect was studied with one bacterial strain, that, although a typical pathogen of implant-related infection, may not be representative of other microorganisms’ susceptibility to the coating;
- The bacterial load used in this study was high and may not reflect the clinical setting, where much lower bacterial contamination is expected, at least in clean surgery. However, it may be speculated that the protective action of the coating may still be more beneficial to prevent implant colonization in the presence of lower contamination;
- Only two concentrations of vancomycin were tested. Although a small dose-dependent effect was observed, no final conclusion may be drawn on the basis of the present study as to regard the best antibiotic concentration to be used in the hydrogel, while other antibacterial drugs may be more indicated, in particular, cases in the clinical setting;
- This study focused on acute colonization of an implant, and data were collected after seven days from surgery. Long-term efficacy of the hydrogel on the colonizing pathogens was not investigated in the present study. A long-term study in rabbits at 28 days is on-going.

In conclusion, the present study provides evidence for the first time that DAC®, a resorbable hydrogel coating, composed of covalently linked hyaluronan and poly-D,L-lactide, which can be hydrated with different antibacterial compounds (vancomycin, tobramycin, gentamicin, meropenem, levofloxacin, linezolid, daptomycin, N-acetylcysteine, etc.) is able to quickly deliver vancomycin locally. Vancomycin-loaded DAC® coating significantly reduces acute local and systemic bacterial count following high local MRSA contamination of an intra-medullary nail, in an animal model, without detectable side effects. Moreover, DAC® hydrogel long-term histocompatibility with bone tissue has been shown. Antibacterial-loaded DAC® coating may represent a possible option to protect orthopaedic implants from bacterial colonization, provided that further studies will confirm its efficacy in vivo and in the clinical setting.
